# Human Periodontal Ligament Derived Progenitor Cells: Effect of STRO-1 Cell Sorting and Wnt3a Treatment on Cell Behavior

**DOI:** 10.1155/2014/145423

**Published:** 2014-04-28

**Authors:** Xiang-Zhen Yan, Sanne K. Both, Pi-Shan Yang, John A. Jansen, Jeroen J. J. P. van den Beucken, Fang Yang

**Affiliations:** ^1^Department of Periodontology and Biomaterials, Radboud University Medical Center, 309 Dentistry, P.O. Box 9101, 6500 HB Nijmegen, The Netherlands; ^2^Department of Periodontology, Shandong University, Jinan, Shandong 250012, China

## Abstract

*Objectives.* STRO-1 positive periodontal ligament cells (PDLCs) and unsorted PDLCs have demonstrated potential for periodontal regeneration, but the comparison between unsorted cells and the expanded STRO-1 sorted cells has never been reported. Additionally, Wnt3a is involved in cell proliferation thus may benefit *in vitro* PDLC expansion. The aim was to evaluate the effect of STRO-1 cell sorting and Wnt3a treatment on cell behavior of human PDLCs (hPDLCs). *Materials and Methods.* STRO-1 positive hPDLCs were sorted and the sorted cells were expanded and compared with their unsorted parental cells. Thereafter, hPDLCs were treated with or without Wnt3a and the cell proliferation, self-renewal, and osteogenic differentiation were evaluated. *Results.* No differences were measured between the expanded STRO-1-sorted cells and unsorted parental cells in terms of proliferation, CFU, and mineralization capacity. Wnt3a enhanced the proliferation and self-renewal ability of hPDLCs significantly as displayed by higher DNA content values, a shorter cell population doubling time, and higher expression of the self-renewal gene *Oct4*. Moreover, Wnt3a promoted the expansion of hPDLCs for 5 passages without affecting cell proliferation, CFU, and osteogenic capacity. *Conclusions.* Expanded STRO-1-sorted hPDLCs showed no superiority compared to their unsorted parental cells. On the other hand, Wnt3a promotes the efficient hPDLC expansion and retains the self-renewal and osteogenic differentiation capacity.

## 1. Introduction

Periodontitis is a multifactorial disease caused primarily by dental plaque microorganisms [1]. Periodontitis is characterized by the destruction of the periodontium, including gingiva, periodontal ligament (PDL), cementum, and alveolar bone. Without adequate treatment, periodontitis will finally lead to tooth loss, which often affects nutrition intake and self-confidence. Approximately 48% of adults have chronic periodontitis and advanced periodontitis is more prevalent among the older age groups [1, 2]. Current treatments are generally successful in preventing active disease, but the regeneration of the lost tissues remains a challenge. Recently, substantial progress has been made in periodontal tissue regeneration by cytotherapeutic approaches to overcome the limitations of existing procedures [3–5].

Several cell types have been used for periodontal regeneration including periodontal ligament cells (PDLCs), bone marrow stromal cells (BMSCs), alveolar periosteal cells (APCs), dental follicle cells (DFCs), and dental pulp cells (DPCs) [3, 5–7]. Tsumanuma et al. transplanted PDLCs, BMSCs, and APCs in canine one-wall intrabony defects for eight weeks [6] and results showed that significantly more newly formed cementum and well-oriented PDL fibers were formed in the PDLCs group than in the other groups. Besides, in an organ culture study performed on tooth root surfaces, new alveolar bone and PDL-like tissues were formed only by PDLCs but not by DFCs, DPCs, or BMSCs [7]. These results indicate that PDLCs may be the most suitable cell source for periodontal tissue regeneration.

STRO-1, one of the most well-known mesenchymal stem-cell markers, has gained increasing interest in stem cell sorting over the past decade [7–11]. For instance, STRO-1 has been utilized for the selection of PDL stem cells [8], dental pulp stem cells [7, 9], and adipose-derived stem cells [10]. STRO-1 positive PDL stem cells are usually utilized for research purpose and their potential to regenerate periodontal tissues* in vivo* has been reported [8]. Since PDLCs contain subpopulations of stem cells [12], the heterogeneous unsorted PDLCs have also been shown to promote periodontal tissue formation [5, 7, 9, 10]. The sorted stem cells in high purity might provide a better cell source for therapeutic purposes compared with the heterogeneous unsorted cells. But STRO-1 positive cells are usually found in low numbers [13, 14] and therefore* in vitro* expansion is needed. However, the expression of STRO-1 was gradually lost during culture expansion, as suggested in previous studies [13, 14]. Yet, the comparison between unsorted parental cells and the expanded STRO-1 sorted cells (equal expansion as the parental cells) has never been reported. Moreover, from a practical point of view, the cell selection and expansion procedure are time-consuming. Thus, it is of importance to compare unsorted parental cells and the expanded STRO-1 sorted cells from PDLCs in order to benefit their future clinical applications.

Along with the high quality, large quantity of cells is necessary for effective therapeutic applications. For instance, 160 million cells would be required for 20 cubic centimeter of tissue engineered bone implant based on using 8 million cells/cm^3^ scaffold [15, 16] to gain substantial bone formation. PDLCs are easily accessible but the cell number is very limited from primary cell culture, and hence it requires* in vitro* expansion before clinical applications. Yet characteristic changes of PDLCs have been observed during passaging [13]. Alkaline phosphatase (ALP) activity of PDLCs gradually decreased as the passage number increased [13]. Thus, finding a method that can benefit the efficient* in vitro* expansion of PDLCs is required.

The cellular signaling pathways that control the proliferation of PDLCs are unclear. A promising candidate is the Wnt signaling, which is involved in tooth development [17, 18]. Wnt signaling affects tooth size [17] and induces continuous tooth generation in mouse [18]. As reported in a previous study [19], in a continuously erupting tooth, cells with the highest level of Wnt responsiveness also show the highest proliferation. Wnt3a, a representative canonical Wnt member, has been recently isolated as an active Wnt molecule [20]. Wnt3a can enhance clonal outgrowth of neural stem cells [21] and promote long-term expansion of mammary stem cells [22]. Furthermore, Wnt signaling has been suggested to play an essential role in osteogenesis* in vitro* and* in vivo *[23–25]. For example, Wnt3a suppressed osteogenic differentiation of mesenchymal stem cells (MSCs) [23]. However, whether Wnt3a can directly control the proliferation and differentiation of adult human PDLCs is not known.

The aim of this study was to evaluate the effect of STRO-1 cell sorting and Wnt3a treatment on cell behavior of human PDLCs (hPDLCs). To this end, STRO-1 positive PDLCs were sorted and then the sorted cells were expanded and compared with their unsorted parental cells in terms of proliferation, colony forming unit (CFU), and mineralization. Thereafter, hPDLCs were treated with or without Wnt3a and the cell proliferation, self-renewal, and osteogenic differentiation were evaluated. It was hypothesized that Wnt3a can benefit the efficient* in vitro* expansion of hPDLCs by enhancing cell proliferation and self-renewal, while inhibiting osteogenic differentiation.

## 2. Materials and Methods

### 2.1. Cell Isolation

All experiments were done by following national guidelines for working with human materials (Dutch federation of biomedical scientific societies. Human tissue and medical research: code of conduct for responsible use, available at: http://www.federa.org/). After patients had signed informed consent, adult hPDLCs from 3 patients (20, 24, and 33 years old, resp.) were obtained from healthy impacted third molars, which were routinely extracted for the prevention of third molar-related morbidity. The PDL was scraped from the middle third of the roots, dissected, and placed in a 25 cm^2^ culture flask in proliferation medium, which contained *α*-MEM (Gibco) supplemented with 10% FCS (Gibco) and 100 units/mL pen/strep (Gibco). Bone was harvested from the posterior maxilla of 1 patient (41 years old) during dental implant surgery. Bone debris was retrieved from the drill surface and chopped into small pieces and put into a 50 mL tube. The tube with minced bone debris and proliferation medium was shaken vigorously and medium with human bone marrow stromal cells (hBMSCs) was collected and plated in a 25 cm^2^ culture flask. Upon 70–80% confluency, hPDLCs and hBMSCs were subcultured for 3 passages and afterwards cryopreserved in *α*-MEM containing 20% FCS and 10% DMSO (Sigma).

### 2.2. Cell Characterization

After defrosting, the morphology of hPDLCs was observed by an inverse phase contrast microscope (Leica DMIL, Germany) at ×10 magnification. The self-renewal and osteogenic ability of hPDLCs from the 4th passage were characterized by colony-forming unit (CFU) efficiency, ALP activity, and mineralization ability (Von-Kossa staining). CFU assay was performed on day 10 in proliferation medium. ALP activity of hPDLCs was measured on day 8 in osteogenic medium (proliferation medium containing 50 *μ*g/mL ascorbic acid, 10 mM sodium**β**-glycerophosphate, and 10 nM dexamethason, all from Sigma). Human fibroblasts from foreskin (a kind gift from Department of Orthodontics and Oral Biology, Radboud University Medical Centre) and hBMSCs were used as negative and positive control for ALP activity assay. Von-Kossa staining was performed after 30 days of culture in osteogenic medium. All the assays are described in detail below.

### 2.3. STRO-1 Fluorescence-Activated Cell Sorting and Testing

The cryopreserved hPDLCs from 2 donors were defrosted and performed for cell sorting experiments. Single hPDLC suspensions containing 10 × 10^6^ cells from the 4th passage were obtained through a 70 *μ*m cell strainer (Falcon BD, Franklin lakes, NJ, USA). Next, the cell suspensions were centrifuged at 400 g for 10 min, resuspended in 1 mL PBS/1%BSA, and preincubated for 20 min on ice. Subsequently, cells were incubated with 200 *μ*L mouse anti-human monoclonal STRO-1 IgM primary antibody (2.5 *μ*g/10^6^ cells, R&D Systems, Minneapolis, MN) in PBS/1%BSA for 15 min on ice and washed 3 times with PBS followed by centrifugation at 250 g for 5 minutes. The cell pellet was resuspended in 200 *μ*L PBS/1%BSA with 10 *μ*L phycoerythrin- (PE-) conjugated goat anti-mouse IgM antibody (R&D Systems, Minneapolis, MN) for 30 min on ice, washed for 3 times, resolved in 400 *μ*L of PBS, and kept on ice until sorting. For blank control, PBS was substituted for the primary and second antibodies. To confirm the specificity of primary antibody binding, nonspecific mouse IgM isotype control (lambda monoclonal, abcam) which ideally matches the primary antibody's host species was substituted for the primary antibody. All incubations were performed in the dark at 4°C. Cells were sorted using a FACStar Plus flow cytometer (Beckton Dickinson & Co., Mountain View, CA). Positivity was defined as a level of fluorescence greater than 99% of the blank (without 1st and 2nd antibodies) and negative (without 1st antibody) control. To prove the efficacy of the applied FACS sorting, sorted cells and controls (STRO-1^+^/STRO-1^−^/un-sorted cells) were evaluated by fluorescence microscopy and flow cytometry. STRO-1^+^ cells were collected and expanded to 10 × 10^6^cells in proliferation medium. The expanded cells were compared with their unsorted parental cells in terms of proliferation (DNA content on days 2, 4, and 6 in proliferation medium), CFU ability (day 10 in proliferation medium), and mineralization capacity (calcium content on day 30 in osteogenic medium). All the assays are described in detail below.

### 2.4. Direct Effect of Wnt3a on hPDLCs

Based on the STRO-1 cell sorting results, unsorted hPDLCs at 4th passage were used in this study. hPDLCs from 3 donors were treated with or without 50 ng/mL Wnt3a (R&D Systems). The dosage was chosen according to a previous study in which 50 ng/mL Wnt3a promoted long-term expansion of mammary stem cells [22]. Then, DNA content, gene expression (self-renewal gene markers* Oct4*,* Nanog* and* Sox2*, and osteogenic gene markers* ALP*,* Runx-2*, and* OC*), ALP activity, and calcium content were evaluated. All the assays are described in detail below.

### 2.5. Functionality of PDLCs Pretreated with Wnt3a

For a long-term study, hPDLCs from passage 4 were subcultured with or without 50 ng/mL Wnt3a up to passage 9, and at each passage the cell doubling time was calculated. After pretreatment with or without Wnt3a for 5 passages, the functionality of Wnt3a-pretreated and control cells was compared in terms of proliferation (DNA content), CFU, and osteogenic differentiation (ALP activity and calcium content). All the assays are described in detail below.

### 2.6. DNA Assay

After 2, 4, and 6 days of incubation in proliferation medium, samples were prepared by washing the cells layers twice with PBS and adding 1 mL of MilliQ to each well, after which repetitive freezing (−80°C) and thawing (37°C) cycles were performed. DNA analysis was performed via a PicoGreen dsDNA quantification kit (Molecular Probes, Leiden, The Netherlands) following manufacturer's instructions. Briefly, 100 *μ*L of DNA standard or sample was incubated with 100 *μ*L of working solution for 10 min at RT in the dark. After incubation, DNA content was measured using a fluorescence microplate reader (Bio-Tek Instruments, Abcoude, The Netherlands) with excitation filter 485 nm and emission filter 530 nm.

### 2.7. ALP Activity

Cells in osteogenic medium were harvested at days 7 and 10 in the same way as cells for the DNA content. Then, 100 *μ*L of substrate solution (p-nitrophenyl phosphate) was added to 20 *μ*L of buffer (0.5 M 2-amino-2-methyl-1-propanol) and 80 *μ*L of sample or standard in a 96-well plate. The standards were made by serial dilutions of 4-nitrophenol at final concentrations of 0–25 nM. The plate was incubated at 37°C for 1 hour. The reaction was terminated by adding 100 *μ*L of 0.3 M NaOH. The absorbance of each well was measured in an ELISA microplate reader (Bio-Tek Instruments, Abcoude, The Netherlands) at 405 nm. ALP activity was normalized to the amount of DNA.

### 2.8. Calcium Content

After 30 days of culture in osteogenic medium, cells were washed twice with PBS and then 1 mL of 0.5 N acetic acid was added to the each well. The tissue culture plate was incubated overnight on a shaking table. The calcium content was measured by the ortho-cresolphthalein complexone (OCPC) method (Sigma). For the biochemical assay, 10 *μ*L samples or standards were incubated with 300 *μ*L of working solution (Genzyme Diagnostics, Cambridge, MA, USA) in a 96-well plate. Standards (0–100 *μ*g/mL) were generated using a CaCl_2_ stock solution. The plate was incubated at RT for 10 min and then the absorbance of each well was measured in the ELISA microplate reader (Bio-Tek Instruments, Abcoude, The Netherlands) at 570 nm.

### 2.9. Von Kossa Staining

After 30 days of osteogenic induction, cells were fixed in 10% formalin for 20 min, rinsed with MilliQ, and then stained with 5% silver nitrate (AgNO_3_, Merck) for 30 min. After washing with distilled water, the staining was developed with 5% sodium carbonate (Na_2_CO_3_, Merck), fixed with 5% sodium thiosulphate (Na_2_S_2_O_3_, Merk), and examined by using a stereomicroscope (Leica MZ12, Germany).

### 2.10. CFU Assay

Single cell suspensions (1000 cells/mL; 100 *μ*L) were seeded into one well of a 6-well plate in proliferation medium. After 10 days, the samples were fixated with 10% formalin and stained with 0.1% toluidine blue (Sigma, Chemical Co., St. Louis, MO, USA). Colony-forming efficiency (an aggregate of ≥50 cells was scored as a colony) was determined by the number of colonies relative to the total number of seeded cells in each plate using a microscope (Leica DMIL, Germany).

### 2.11. Real-Time PCR

Cells in proliferation medium at day 5 (*Oct4, Nanog, *and* Sox2* gene expression) and in osteogenic medium at days 7 and 14 (*ALP, Runx-2, *and* OC *gene expression) were washed twice with PBS. Total RNA was extracted using TRIzol reagent (Invitrogen, Breda, The Netherlands) following manufacturer's instructions. RNA concentrations and purity were determined by NanoDrop (ND-2000, Thermo Scientific). Then, the reverse transcriptase (RT) reaction of 1 *μ*g RNA for each sample was performed using the Superscript III First-strand Synthesis System (Invitrogen, Breda, the Netherlands) for RT-PCR. The cDNA was amplified and gene expression was quantified with real-time PCR (BIORAD, CFX96 real-time system). The primers used were* Oct4, Nanog, Sox2, ALP, Runx-2, *and* OC *(sequences in Table 1). The expression levels were analyzed versus the housekeeping gene* GAPDH*. The specificity of the primers was tested before the real-time PCR reaction. IQ SYBR Green Supermix PCR kit (BioRad, Hemel Hempstead, United Kingdom) was used for real-time measurement. The melting temperature (Tm) employed for each primer pair was 60°C. The gene expression was calculated using the 2^−ΔΔCt^ method and the control was used as the calibrator group.

### 2.12. Statistical Analysis

Each assay was performed in triplicate for each donor and statistical analysis was performed using GraphPad InStat (GraphPad Software, San Diego California USA). Results were statistically evaluated for each donor using an unpaired* t-*test (significance level,* P* < 0.05).

## 3. Results

As all the donors showed the similar trend, only the result from one donor was presented below unless specifically mentioned.

### 3.1. Characterization of hPDLCs

**Figure 1:**
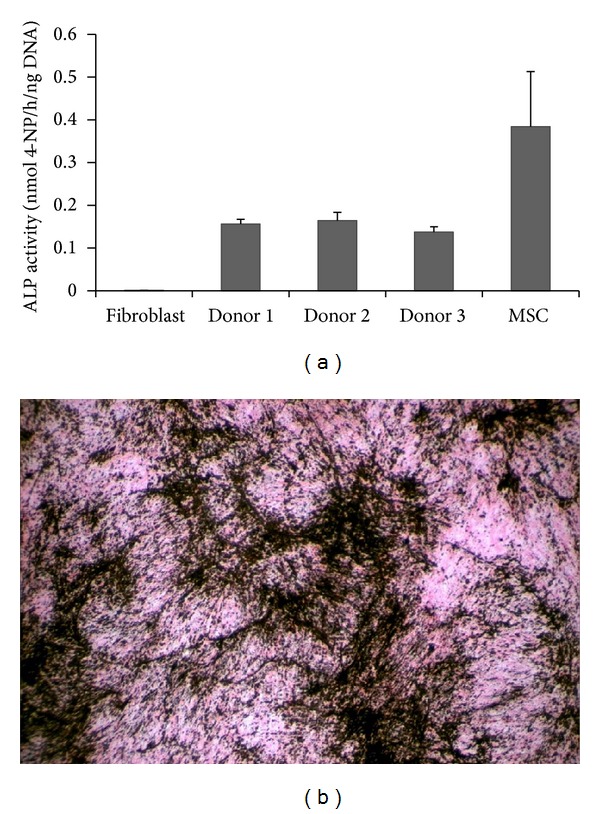
Characterization of hPDLCs. (a) ALP activity of the hPDLCs was lower compared to hBMSCs (positive control) on day 8, while fibroblasts were negative. (b) Mineral deposition was detected by a Von Kossa staining (black color) after 30 days of osteogenic induction. Error bars represent standard deviation.

hPDLCs were characterized on basis of their morphology, self-renewal, and osteogenic ability. Microscopy observation revealed spindle-shape cell morphology. hPDLCs displayed the ability to form colonies when cultured in proliferation medium after 10 days. ALP activity was confirmed in the hPDLC culture after 8 days in osteogenic medium. The ALP activity in hPDLCs was in between hBMSCs (positive control) and fibroblasts (negative control) cultured at the same conditions (Figure 1(a)). hPDLCs induced mineralization as determined by Von Kossa staining after 30 days of osteogenic induction (Figure 1(b)).

### 3.2. Comparison between Expanded STRO-1-Sorted Cells and Unsorted Parental Cells

**Figure 2:**
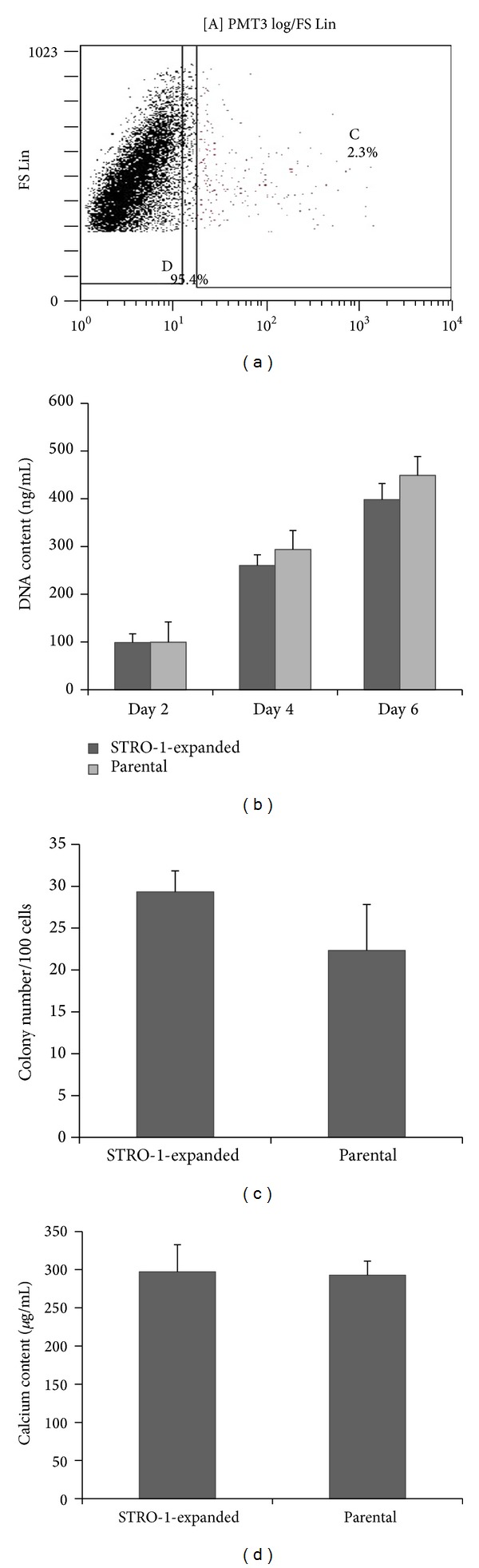
Representative results of STRO-1 cell sorting. (a) The percentage of sorted STRO-1^+^ cells decreased to 2.3% after 4 passages of* in vitro* expansion. (b) No difference in proliferation was observed between the expanded STRO-1-sorted cells and the unsorted parental cells. (c) Expanded STRO-1-sorted cells displayed no difference compared to the unsorted parental cells in colony forming number. (d) No difference in calcium content was observed between expanded STRO-1-sorted cells and parental hPDLCs. Error bars represent standard deviation.

The efficiency of FACS sorting was proved by fluorescent microscopy observation and flow cytometry. The STRO-1^+^ cells were expanded for 4 passages to achieve 10 × 10^6^ cells, the same number as their unsorted parental cells. During this expansion, the percentage of STRO-1^+^ cells decreased significantly from 95.3% to 2.3% (Figure 2(a)). Additionally, after 4 passages of* in vitro* expansion, no significant difference was observed between the expanded STRO-1-sorted cells and unsorted parental cells in terms of DNA content, CFU number, and calcium content (Figures 2(b)–2(d)).

### 3.3. The Direct Effect of Wnt3a on hPDLCs

**Figure 3:**
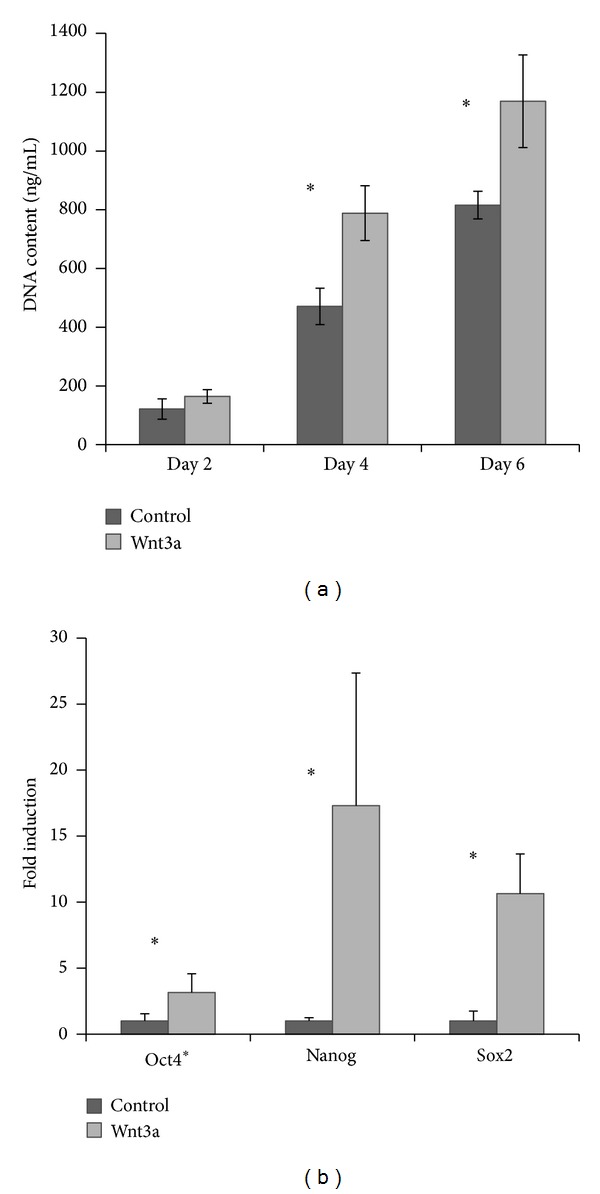
Representative results showed Wnt3a promoted proliferation and self-renewal ability of hPDLCs. (a) Wnt3a-treated cells displayed a significant increase in DNA content compared to untreated cells cultured in proliferation medium. (b) Wnt3a-treated cells exhibited a higher expression of* Oct4*,* Nanog*, and* Sox2* after 5 days of culture. *P < 0.05; error bars represent standard deviation.

The effect of Wnt3a on hPDLC proliferation was assessed by measuring cell DNA content and calculating cell doubling time during passaging. Wnt3a treated cells exhibited a significant increase (*P *< 0.05; around 50% increase for day 4 and day 6 in DNA content compared to the nontreated control group; Figure 3(a)). In addition, Wnt3a-treated cells displayed significantly shorter population doubling time when compared to untreated control cells, which were 25.46 ± 3.76 versus 30.73 ± 0.9 hours (*P* < 0.05).

The self-renewal ability was evaluated by the expression of the self-renewal genes octamer-binding transcription factor 4 (*Oct4*),* Nanog,* and sex determining region Y-box 2 (*Sox2*). The mRNA expression of* Oct4* was significantly higher (*P *< 0.05) for the cells treated with Wnt3a than for the control cells (Figure 3(b)). Wnt3a-treated cells also exhibited higher* Nanog *and* Sox2* expression, but a significant difference was only observed in one donor.

**Figure 4:**
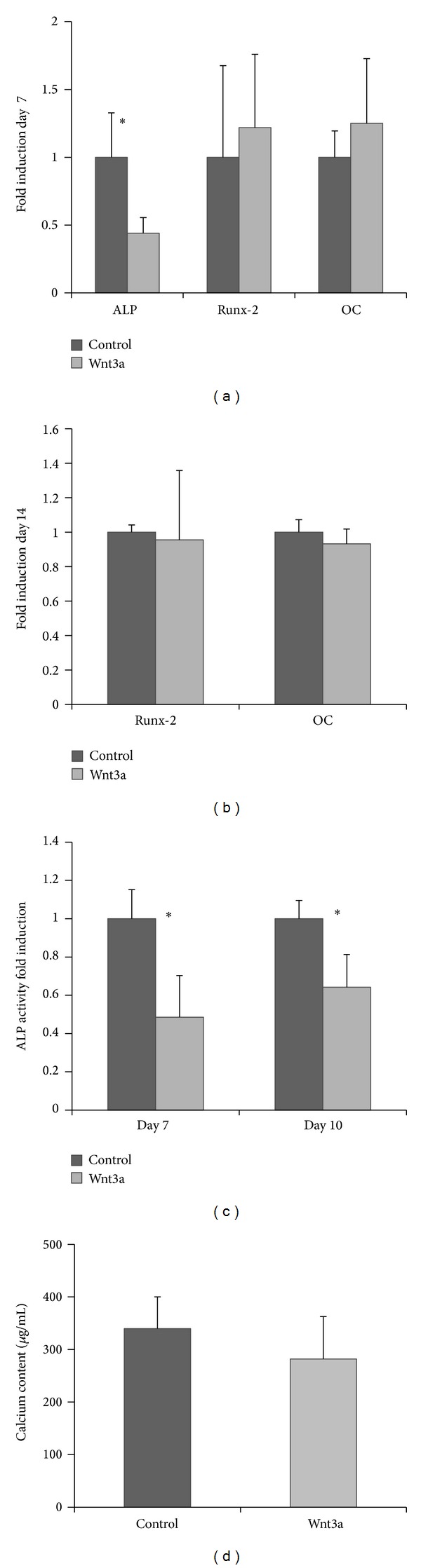
Representative results showed Wnt3a delayed osteogenic differentiation of hPDLCs. (a) ALP mRNA was downregulated by Wnt3a after 7 days of osteogenic induction. (b) The gene expression was equal for* Runx-2* and* OC* between two groups. (c) ALP activity was downregulated by Wnt3a on days 7 and 10. (d) Wnt3a has no effect on the calcium content after 30 days of osteogenic induction. *P < 0.05; error bars represent standard deviation.

Osteogenic differentiation was assessed by gene expression of* ALP, *runt-related transcription factor 2 (*Runx-2*), and osteocalcin (*OC*), ALP activity, calcium content in the extracellular matrix, and Von Kossa staining. PCR revealed significantly lower (*P *< 0.05)* ALP* gene expression for Wnt3a-treated cells at day 7 compared to the control group (Figure 4(a)). For the* Runx-2 *and* OC* gene expression no significant difference was found at day 7 and day 14 between the Wnt3a-treated and control groups (Figures 4(a) and 4(b)). ALP activity at days 7 and 10 demonstrated significantly lower (*P *< 0.05) levels for Wnt3a-treated cells versus untreated cells (Figure 4(c)). The long-term mineralization was evaluated by calcium content deposited by the cells and a Von Kossa staining after 30 days of culture. Both Wnt3a treated and untreated cells induced mineralization as determined by Von Kossa staining and no significant difference in calcium content was observed between the two groups (Figure 4(d)).

### 3.4. Functionality of Wnt3a Pretreated hPDLCs

**Figure 5:**
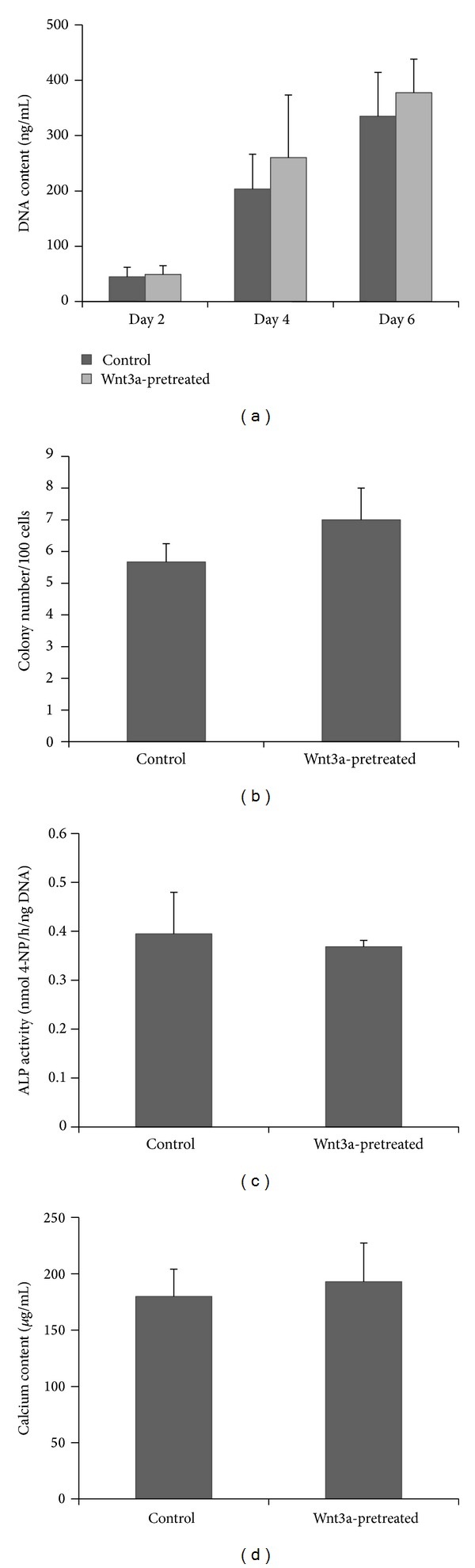
Representative results showed Wnt3a pretreatment did not affect functionality of hPDLCs after long-term expansion. (a) No difference in DNA content was observed between Wnt3a-pretreated cells and control cells after 5 passages. (b) Wnt3a-pretreated cells displayed no difference compared to the control cells in CFU number. (c) No difference in ALP activity was observed between Wnt3a-pretreated cells and control cells. (d) No difference in calcium content was detected between Wnt3a-pretreated cells and control cells after 5 passages. Error bars represent standard deviation.

After passage 9, Wnt3a was no longer added to the culture medium and the proliferation, CFU, and osteogenic differentiation capacity were investigated to evaluate whether the functionality of Wnt3a-pretreated cells was affected by the shorter cell doubling time. The results showed that both Wnt3a-pretreated cells and control cells showed abilities to proliferate, form colonies, and osteogenically differentiate. In addition, no difference was observed between Wnt3a-pretreated cells and control cells in terms of DNA content (Figure 5(a)), CFU number (Figure 5(b)), ALP activity (Figure 5(c)), or calcium content (Figure 5(d)).

## 4. Discussion

The aim of the current study was to evaluate the effect of STRO-1 cell sorting and Wnt3a treatment on cell behavior of hPDLCs. The characteristics of unsorted parental cells and expanded STRO-1-sorted cells were compared to find the appropriate PDL cell population for clinical applications. The effect of Wnt3a on proliferation, self-renewal, and osteogenesis of hPDLCs was evaluated to test the potential of Wnt3a for* in vitro* expansion of hPDLCs. No differences were measured between the expanded STRO-1-sorted cells and unsorted parental cells in terms of proliferation, CFU, and mineralization capacity. Wnt3a promoted the proliferation and self-renewal ability of hPDLCs, as displayed by an increased DNA content, shorter population doubling time, and higher expression of* Oct4*. Wnt3a also stimulated the efficient* in vitro* expansion of hPDLCs for at least 5 passages without affecting cell functionalities in terms of proliferation, CFU, and osteogenic capacity.

STRO-1 positive cells are usually found in low numbers [13] and therefore* in vitro* expansion is needed. Our results showed that the percentage of STRO-1 positive cells decreased significantly during culture expansion. Additionally, after 4 passages of* in vitro* expansion, there were no differences in proliferation, CFU ability, and mineralization capacity between sorted and initially unsorted cells. A similar phenomenon has been observed with hBMSCs (unpublished data): the STRO-1 sorted hBMSCs returned to the same state as their unsorted parental cells only in 7 days of* in vitro* expansion. Based on the results and also the time-consuming procedure of the cell selection and expansion, unsorted primary cells may provide a better cell source for future clinical applications. Therefore, unsorted hPDLCs were used in the current study to test the effect of Wnt3a.

The outcome of DNA content indicates that Wnt3a enhances the proliferation of hPDLCs significantly. Additionally during the long-term study, the cells were treated with or without Wnt3a for 5 passages with the same cell seeding density. Wnt3a-treated cells showed more cell doublings than the control group at each passage, indicating the enhancement of Wnt3a on the proliferation of hPDLCs.* Oct4*,* Nanog*, and* Sox2* are key regulators essential for self-renewal of pluripotent cells [26]. These self-renewal factors are also expressed in hPDLCs [12, 27] and thus they were chosen in this study. Based upon the results, the self-renewal ability of hPDLCs was promoted by Wnt3a as displayed by the higher expression of self-renewal gene expressions. This is consistent with one previous study in which self-renewal and gene expression were stimulated in embryonic stem cells by adding Wnt3a [28]. These results do suggest that Wnt3a may benefit the* in vitro* expansion of primary cells for further cell-based clinical applications.

Our results further suggest that Wnt3a mediates some aspects of osteogenic differentiation. ALP mRNA-levels and ALP-activity were reduced significantly by Wnt3a during osteogenic differentiation. On the other hand, it has to be noted that for the mineralization, no effect of Wnt3a was observed after 30 days of culture, indicating Wnt3a may have a delayed effect on osteogenic differentiation of hPDLCs. The effect of Wnt signaling on the STRO-1^+^ cells in the efficiency of osteogenic differentiation was not evaluated here. Since cells selected by mesenchymal stem-cell marker STRO-1 are considered to be in an undifferentiated state, it can be speculated that enhanced activation of canonical Wnt signaling could also inhibit osteogenic differentiation of STRO-1^+^ cells based on the previous study, where it has been demonstrated that enhanced activation of canonical Wnt signaling hindered osteogenic differentiation of undifferentiated MSCs. Interestingly, some* in vivo* studies reported that Wnt3a could enhance implant osseointegration [25] and promote a better healing of a calvarial defect in adult mice [24]. The enhancement of proliferation or self-renewal by Wnt3a may explain the acceleration of osteogenesis* in vivo*.

Recently Yamada et al. reported that XAV939, an inhibitor of canonical Wnt signaling, inhibited ALP activity of hPDL-derived MSCs, indicating the promoting effect of canonical Wnt signaling pathway during the osteogenic differentiation of hPDLCs [29]. However, this mechanism is not consistent with the findings from this and also the other studies [23, 30]. This discrepancy might be caused by the complexity of Wnt signaling. It has been reported that activation of canonical Wnt signaling may bring out opposite biological activity in the context of osteogenic differentiation depending on the threshold levels of its activation, the status of cell, and Wnt ligands concentration [24]. Therefore, the comprehensive understanding of the roles of both appropriate dose concentrations of Wnt3a treatment and endogenous activity of canonical Wnt signaling will be needed in further studies.

More hPDLCs with higher self-renewal ability can be obtained by using Wnt3a. To further confirm the functionality of Wnt3a expanded hPDLCs, we subcultured hPDLCs from passage 4 with or without Wnt3a up to passage 9 and compared the functionalities of Wnt3a-pretreated and control cells. Wnt3a promoted the rapid* in vitro* expansion of hPDLCs for at least 5 passages, as displayed by the shorter population doubling time, without interfering with their functionalities compared to untreated control cells. Wnt3a pretreatment resulted in much more hPDLCs with similar proliferation, CFU, and osteogenic capacity as the untreated control cells, which is of importance for the clinical use of hPDLCs because a large number of cells are required for cytotherapeutic purposes. In a previous study, Zeng and Nusse reported that Wnt3a could promote long-term expansion of mammary stem cells and maintain their self-renewal ability [22]. These data indicate that Wnt3a may be used as a useful culture supplement for the* in vitro* expansion of hPDLCs to benefit their clinical application.

## 5. Conclusions

The proliferation, CFU, and mineralization capacity of parental hPDLCs and expanded STRO-1-sorted cells were compared to find the appropriate PDL cell population for future clinical applications. The results provide evidence that STRO-1-sorted hPDLCs after expansion are not superior compared to their unsorted parental cells, suggesting the time-consuming cell selection and expansion procedure can be avoided in cell-based periodontal regeneration. In contrast, Wnt3a does have an effect on hPDLCs by promoting cell proliferation and self-renewal, as displayed by an increased DNA content, a shorter cell population doubling time, and higher expression of the self-renewal gene* Oct4*. In addition, Wnt3a promoted the efficient* in vitro* expansion of hPDLCs for at least 5 passages without affecting the self-renewal and osteogenic differentiation capacity, indicating its potential for rapid and efficient* in vitro* cell expansion for future clinical applications.

## Figures and Tables

**Table 1:** Primer sequences used for real-time qPCR.

	Forward (5′→3′)	Reverse (5′→3′)
*Oct4 *	TTGCCCTTCTGGCGCCGGTTA	GTCAGGCCCGTCTCAGCTCATTG
*Nanog *	TTGTCCCCAAAGCTTGCCTTGCT	TTCTTACCAGTCTCCGTGTGAGGC
*Sox2 *	AAAAACAGCCCGGACCGCGT	TCGTCGATGAACGGCCGCTT
*ALP *	GGGACTGGTACTCGGATAACGA	CTGATATGCGATGTCCTTGCA
*Runx-2 *	GAGCACAAACATGGCTGAGA	TGGAGATGTTGCTCTGTTCG
*OC *	AGGGCAGCGAGGTAGTGAAGA	TAGACCGGGCCGTAGAAGC
*GAPDH *	CGATGCTGGCGCTGAGTAC	CGTTCAGCTCAGGGATGACC
